# Perceived stress as mediator for longitudinal effects of the COVID-19 lockdown on wellbeing of parents and children

**DOI:** 10.1038/s41598-021-81720-8

**Published:** 2021-02-03

**Authors:** Michelle Achterberg, Simone Dobbelaar, Olga D. Boer, Eveline A. Crone

**Affiliations:** 1 Department of Psychology, Education and Child studies, Erasmus School of Social and Behavioral Sciences, Erasmus University Rotterdam, Rotterdam, The Netherlands; 2 Faculty of Behavioral and Social Sciences, Leiden Consortium Individual Development, Leiden University, Leiden, The Netherlands; 3 Developmental and Educational Psychology, Faculty of Behavioral and Social Sciences, Leiden University, Leiden, The Netherlands

**Keywords:** Human behaviour, Psychology

## Abstract

Dealing with a COVID-19 lockdown may have negative effects on children, but at the same time might facilitate parent–child bonding. Perceived stress may influence the direction of these effects. Using a longitudinal twin design, we investigated how perceived stress influenced lockdown induced changes in wellbeing of parents and children. A total of 106 parents and 151 children (10–13-year-olds) filled in questionnaires during lockdown and data were combined with data of previous years. We report a significant increase in parental negative feelings (anxiety, depression, hostility and interpersonal sensitivity). Longitudinal child measures showed a gradual decrease in internalizing and externalizing behavior, which seemed decelerated by the COVID-19 lockdown. Changes in parental negative feelings and children’s externalizing behavior were mediated by perceived stress: higher scores prior to the lockdown were related to more stress during the lockdown, which in turn was associated with an increase in parental negative feelings and children’s’ externalizing behavior. Perceived stress in parents and children was associated with negative coping strategies. Additionally, children’s stress levels were influenced by prior and current parental overreactivity. These results suggest that children in families with negative coping strategies and (a history of) parental overreactivity might be at risk for negative consequences of the lockdown.

## Introduction

The transition from childhood to adolescence (10–13-years) is known as a period of increased emotional reactivity and social reorientation^1–3^. During this time, parent–child relations may change given that children start to spend more time with peers^4^. However, current measures to reduce the spread of Corona Virus Disease 2019 (COVID-19), such as home lockdown and social distancing, radically reduce adolescents’ opportunities to engage in peer relations outside their household^5,6^. Whereas children and adolescents are largely unaffected by COVID-19 in terms of infectious morbidity and mortality, dealing with lockdown and quarantine may have negative effects on their wellbeing^7^. At the same time, a lockdown situation might also reduce daily hassles and stress in some families, possibly facilitating parent–child bonding^8,9^. One potential mechanism that may influence the direction of wellbeing during lockdown is perceived stress by parents and children.

Prior research has shown that parental distress and parental mental health in disaster situations are associated with increased vulnerability to distress and poor mental health in children and adolescents^10^. We tested the hypothesis that perceived stress influences COVID-19 lockdown induced changes in wellbeing of parents and children (10–13-year-old), using a longitudinal twin design. The current study aimed to answer the following three questions: (1) How does the COVID-19 lockdown affect longitudinal changes in wellbeing of parents and children? (2) How does the level of perceived stress during the lockdown influence longitudinal changes in wellbeing, and (3) Which factors influence the level of perceived stress during the lockdown in parents and children? These questions were examined using the longitudinal twin study of the Leiden Consortium on Individual Development (L-CID^1^). This ongoing longitudinal study involves multiple assessments of wellbeing in twin families and was extended with an online assessment during the COVID-19 lockdown. It therefore provides us with the unique possibility to test for changes across time and specifically in relation to the COVID-19 lockdown.

The recent COVID-19 pandemic has created external stressors in many households, as parents have to juggle between home schooling children, working remotely or being unable to work at all, while worrying about possible financial and health concerns for the family^11^. Given the pandemic nature of COVID-19, this currently affects families all over the world. In periods of external stressors, parents often show an increase in parental negative feelings, i.e., more feelings of depression, anxiety, hostility and interpersonal sensitivity^12,13^. Previous studies reported increased family violence and reactivity during periods of crisis^11,14^. That is, increased stress might lead to frustration, anger and irritability in parents^15^, which is reflected in overreactive parenting^16^. Children exposed to traumatic events such as war and disasters are at high risk for developing posttraumatic stress disorder, disrupted sleep, and emotional and behavioral problems^17,18^. Previous studies on external stressors have thus shown that external stressors can induce stress in families, which in turn might lead to a decrease in wellbeing in both parents and children^9,10,15^. The COVID-19 pandemic, however, might be different in its nature of disaster, as the lockdown regulations could also bring families closer together through increased parent–child bonding and time for reflection^8,9,19^. To shed light on how the COVID-19 lockdown affected wellbeing of parents and children we investigated time-related changes in parental negative feelings and overreactivity, and children’s internalizing and externalizing behavior, using annual measures across the past 5 years.

An important question concerns the consequences of stress levels in parents, as well as the stress levels in children in response to this pandemic, specifically in children in the age range 10 to 13 years who are entering a period of social reorientation^2,4^. Therefore, our second aim was to investigate how perceived stress during the lockdown influenced longitudinal changes in wellbeing of parents and children. To this end, we tested both moderation and mediation models. Changes in wellbeing might be *moderated* by perceived stress: families that experience high levels of stress might show a decrease in wellbeing, whereas families with low perceived stress might show an increase in wellbeing^8^. However, a mediating influence of stress might also be possible. That is, parents and children with decreased wellbeing prior to the lockdown might be more prone to perceive more stress during lockdown, which might result in even lower wellbeing during lockdown^10,20^. Determining the way in which perceived stress influences longitudinal changes in wellbeing is particularly important to detect which families might experience the most negative outcomes of the COVID-19 lockdown.

The extent to which children and parents are sensitive to COVID-19 stress may be dependent on individual differences in coping and reflection^9^. For example, decreased social obligations might provide additional time for reflection, both in parents as well as children. Furthermore, the closing of sport clubs and extracurricular activities might reduce the daily hassles of many families, thereby decreasing stress in some families. Indeed, some children seem to experience alleviation of social and school pressure and enjoy the more intensive family life^8^. Prior research showed that positive coping can be a protector for psychological problems in children^21^. Studies on parents whose children were acutely hospitalized showed that positive coping strategies reduced parental stress and anxiety^22^. Similarly, parents who use positive coping strategies might have relatively low parental stress levels and parental reactivity during COVID-19 lockdown. As such, high levels of positive parental coping and reflection could have positive consequences by increasing family bonding and improve parent–child relations. The third aim of the current study was to investigate factors that influenced the level of perceived stress in parents and children. Based on previous studies^21,22^, we predicted that positive coping strategies (e.g., positive reappraisal) would be related to less perceived stress, whereas negative coping strategies (e.g., rumination) would result in higher perceived stress, both in parents and children. Moreover, we expected that perceived stress in children would additionally be influenced by parental factors, such as parental negative feelings^12^ and parental overreactivity^14^. We used the twin design to our advantage by exploratively testing to what extent children’s stress and coping strategies during the pandemic were influenced by genetic and environmental influences. Previous studies in adults have shown that chronic stress is heritable^23^. Coping styles, on the other hand, have been shown to be influenced by both heritability as well as shared environmental influences^24^.

## Results

### Longitudinal changes due to COVID-19

#### Parental negative feelings and overreactivity

**Figure 1:**
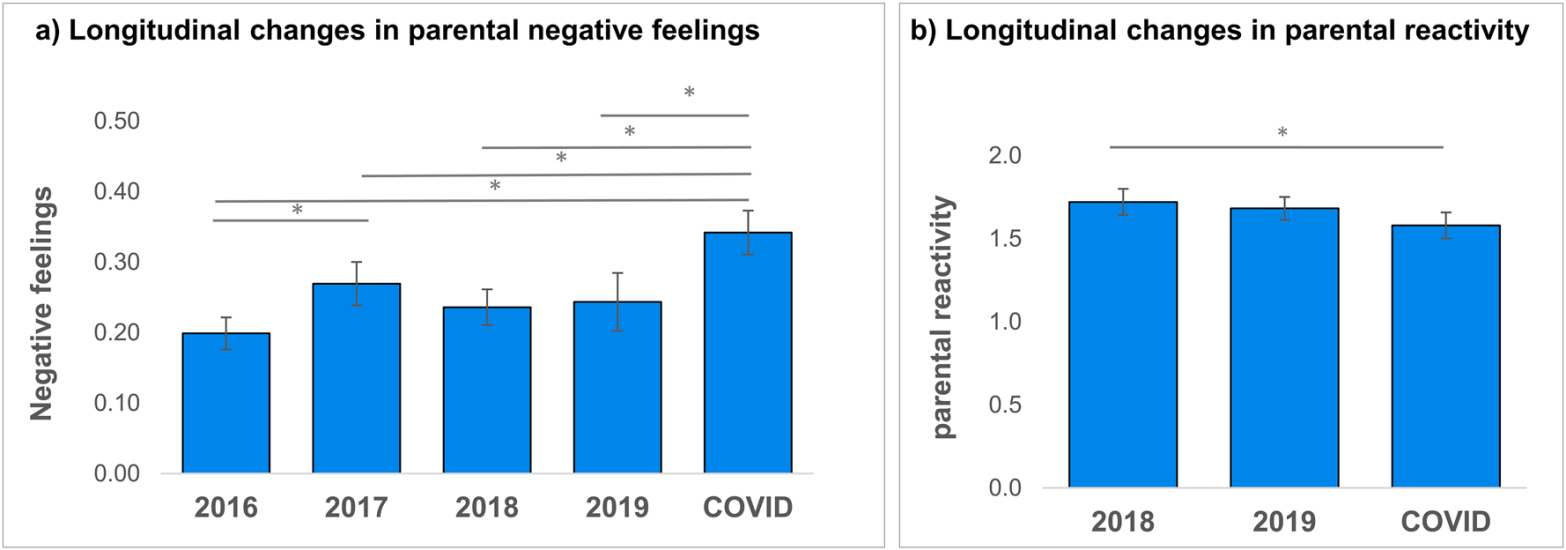
Longitudinal changes in parental negative feelings (**a**) and parental overreactivity (**b**). Asterisk indicate significant differences (*p* < .05).

We investigated whether the COVID-19 lockdown would affect longitudinal changes in parental negative feelings and parental overreactivity, by testing a time-related change during the lockdown relative to before lockdown. Data from the BSI was non-normally distributed at every time point and distributions were skewed to the left (floor effect). Friedman’s test showed a significant main effect of time on parental negative feelings (*χ*^2^(4) = 16.46, *p* = .002, N = 90, see Fig. 1a). These results remained significant after excluding two extreme data points at T4. Bonferroni corrected post-hoc tests showed a significant difference between Tcovid and T1, T3 and T4 (see Table 1). These results suggest a specific increase in parental negative feelings during the COVID-19 pandemic lockdown.

**Table 1:** Z-values for post-hoc pairwise comparisons on longitudinal changes in parental negative feelings (measured with brief symptom inventory subscales anxiety, depression, hostility and interpersonal sensitivity).

	T2	T3	T4	Tcovid
**Parental negative feelings**				
T1	− 2.78*	− 1.17	− 0.31	− 4.15**
T2	–	− 1.01	− 1.58	− 2.42*
T3		–	− 1.25	− 3.82**
T4			–	− 4.15 **

* *p* < .05; ** *p* < .005 (Bonferroni correction).

For parental overreactivity, a repeated measures ANOVA showed a significant main effect of time (*F*(2, 91) = 9.47, *p* = .003, see Fig. 1b). Pairwise comparisons revealed a significant decrease in parental overreactivity between T3 and T_COVID_ (*p* = .008). The decrease between T4 and T_COVID_ showed a similar pattern but was not significant (*p* = .073). These results indicate a gradual decrease in parental overreactivity over time, but specific effects of the COVID-19 pandemic lockdown were not consistent.

#### Children’s internalizing and externalizing behavior

**Figure 2:**
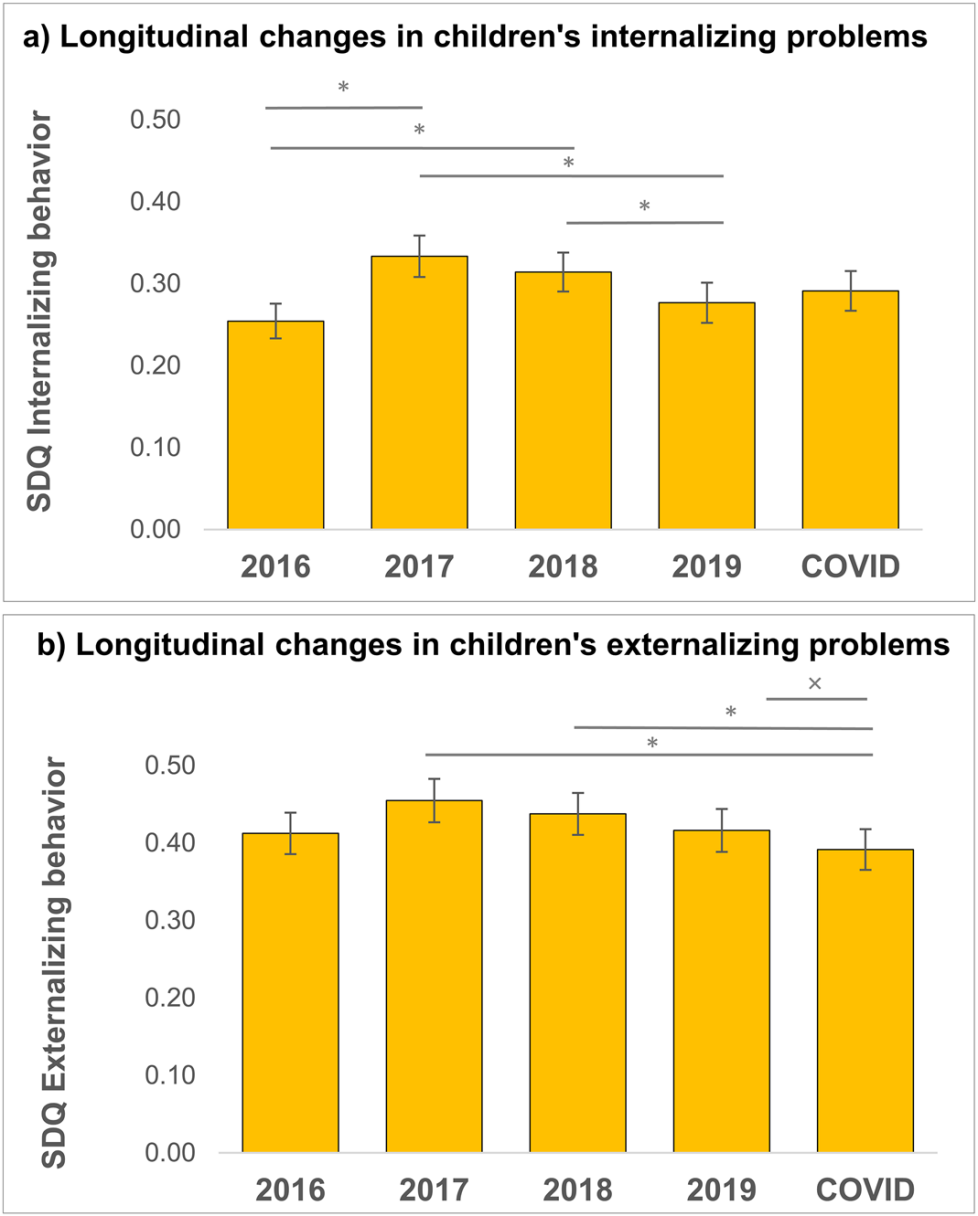
Longitudinal changes in internalizing (**a**) and externalizing (**b**) behavior in children. Asterisk indicate significant differences (*p* < .05), crosses indicate marginally significant differences (*p* = .056).

Second, we investigated whether the COVID-19 lockdown would affect longitudinal changes in children’s internalizing and externalizing behavior reflected by a time-related change in behavior during the lockdown relative to before lockdown. For both subscales, data was non-normally distributed at every time point and distributions were skewed to the left (floor effect). Friedman’s test showed a significant main effect of time on parent reported internalizing behavior (*χ*^2^(4) = 10.65, *p* = .030, N = 179, see Fig. 2a). Follow-up tests showed a significant increase in internalizing behavior between T1 and T2 and between T1 and T3 and a significant decrease in internalizing behavior between T2 and T4 and between T3 and T4 (Table 2). Pairwise comparisons did not survive Bonferroni correction (*α* = .005). There were no significant differences with T_COVID_, suggesting no significant influence of the COVID-19 lockdown on children’s internalizing behavior.

**Table 2:**
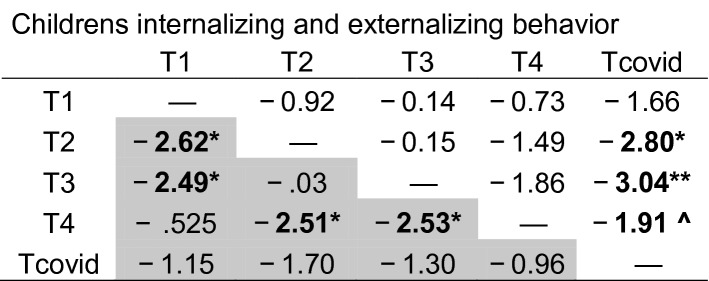
Z values for post-hoc pairwise comparisons on longitudinal changes in children’s internalizing (grey columns) and externalizing (white columns) behavior).

^*p* = .056; *p < .05; **p < .005 (Bonferroni correction).

For externalizing behavior, Friedman’s test showed a significant main effect of time (*χ*^2^(4) = 12.80, *p* = .012, N = 179 see Fig. 2b). Follow-up tests showed a significant decrease in externalizing behavior between T2 and T_COVID_ and between T3 and T_COVID_ (Table 2). Decreases between T4 and T_COVID_ were marginally significant (*p* = .056). These results indicate a decrease in externalizing behavior over time across development, and this decrease is decelerated by the COVID-19 pandemic lockdown.

### Stress during COVID-19 lockdown

**Figure 3:**
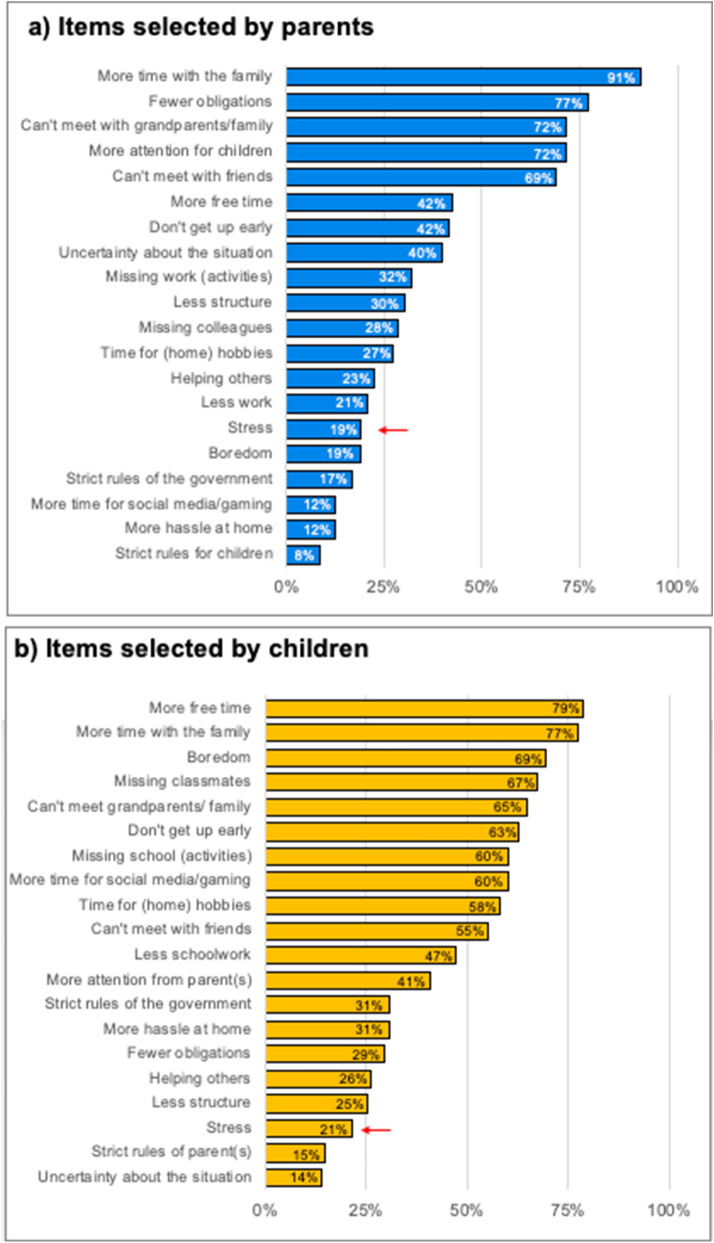
Percentage of selected items that parents (**a**) and children (**b**) selected to apply to them during the past two weeks of the COVID-19 pandemic.

We provided a list of 20 COVID-19 lockdown related items to examine what parents and children were experiencing during the COVID-19 pandemic lockdown. Figure 3 shows the percentage of parents (Fig. 3a) and children (Fig. 3b) that selected each item as to be relevant to them in the past two weeks of COVID-19 lockdown. A large percentage of both parents (91%) and children (77%) selected *“More time with the family*”.

Interestingly, relatively few parents (19%) and children (21%) selected that stress applied to them in the last two weeks of lockdown. Stress was also measured with the Perceived Stress Scale (measured on a 0–4 scale) and ranged between 0 and 2.4 in parents and between 0 and 2.8 in children (Table 3). Perceived stress of children and parents were not significantly correlated (*r* = .10, *p* = .209). We aimed to investigate whether perceived stress was a moderator or mediator for longitudinal changes. We specifically focused on the measures that showed COVID-19 specific changes: parental negative feelings and children’s externalizing behavior.

**Table 3:** Descriptives of questionnaire measures.

Questionnaire descriptives	N	Range	Mean	SD	α
**Brief symptom inventory (BSI, 18 items)**					
T1	97	0.00–1.10	0.20	0.22	0.84
T2	104	0.00–1.90	0.27	0.32	0.89
T3	104	0.00–1.62	0.24	0.26	0.86
T4	101	0.00–2.95	0.24	0.41	0.95
T4*	99	0.00–1.00	0.19	0.22	0.83
T_COVID_	105	0.00–1.43	0.34	0.32	0.86
**Parenting scale—overreactivity (PS, 9 items)**					
T3	94	0.00–3.78	1.72	0.77	0.80/0.80°
T4	98	0.00–3.50	1.68	0.69	0.71/0.71°
Parents—T_COVID_	101	0.00–3.50	1.58	0.78	0.75/0.78°
Children—T_COVID_	148	0.11–4.33	1.95	0.79	0.70
**Strengths and difficulties questionnaire internalizing behavior (SDQ, 6 items)**					
T1	209	0.00–1.33	0.25	0.30	0.74
T2	206	0.00–1.50	0.33	0.36	0.70
T3	200	0.00–1.50	0.31	0.34	0.62
T4	203	0.00–1.83	0.28	0.35	0.75
T_COVID_	209	0.00–1.67	0.29	0.35	0.74
**Strengths and difficulties questionnaire externalizing behavior (SDQ, 6 items)**					
T1	192	0.00–1.83	0.41	0.37	0.69
T2	206	0.00–1.83	0.45	0.40	0.71
T3	200	0.00–1.67	0.44	0.38	0.69
T4	203	0.00–1.67	0.42	0.39	0.74
T_COVID_	209	0.00–1.83	0.39	0.38	0.71
**Perceived stress scale (PSS, 10 items)**					
Parents—T_COVID_	101	0.00–2.40	0.96	0.49	0.79
Children—T_COVID_	150	0.00–2.80	1.13	0.54	0.68
**Cognitive emotion regulation questionnaire, positive coping (CERQ, 6 items)**					
Parents—T_COVID_	106	0.33–4.00	2.11	0.72	0.73
Children—T_COVID_	148	0.17–3.33	1.56	0.70	0.66
**Cognitive emotion regulation questionnaire, negative coping (CERQ, 6 items)**					
Parents—T_COVID_	106	0.00–2.33	0.94	0.51	0.57
Children—T_COVID_	148	0.00–2.50	0.61	0.53	0.74

*Without 2 extremes (mean score 2.40 and 2.95).

°Separate alpha's for questions on child 1 and child 2.

#### Perceived stress as moderator for changes in wellbeing

Parental negative feelings during the lockdown (T_COVID_) were significantly predicted by parental negative feelings prior to the lockdown (T4; *b* = .53, *t* = 2.56, *p* = .012) and by perceived stress during lockdown (*b* = .38, *t* = 7.03, *p* < .001), but not by the interaction between prior parental psychological problems and perceived stress (*b* = − .16, *t* = -1.28, *p* = .211) indicating no significant moderation effect.

Children’s externalizing behavior during the lockdown was significantly predicted by prior externalizing behavior (T4; *b* = .48, *t* = 3.47, *p* < .001). Neither perceived stress during lockdown (*b* = .03, *t* = 0.50, *p* < .620), nor the interaction between prior externalizing behavior and perceived stress (*b* = .13, *t* = 1.36, *p* < .176) were significant predictors for externalizing behavior during lockdown, indicating no significant moderation effect.

#### Perceived stress as mediator for changes in wellbeing

**Figure 4:**
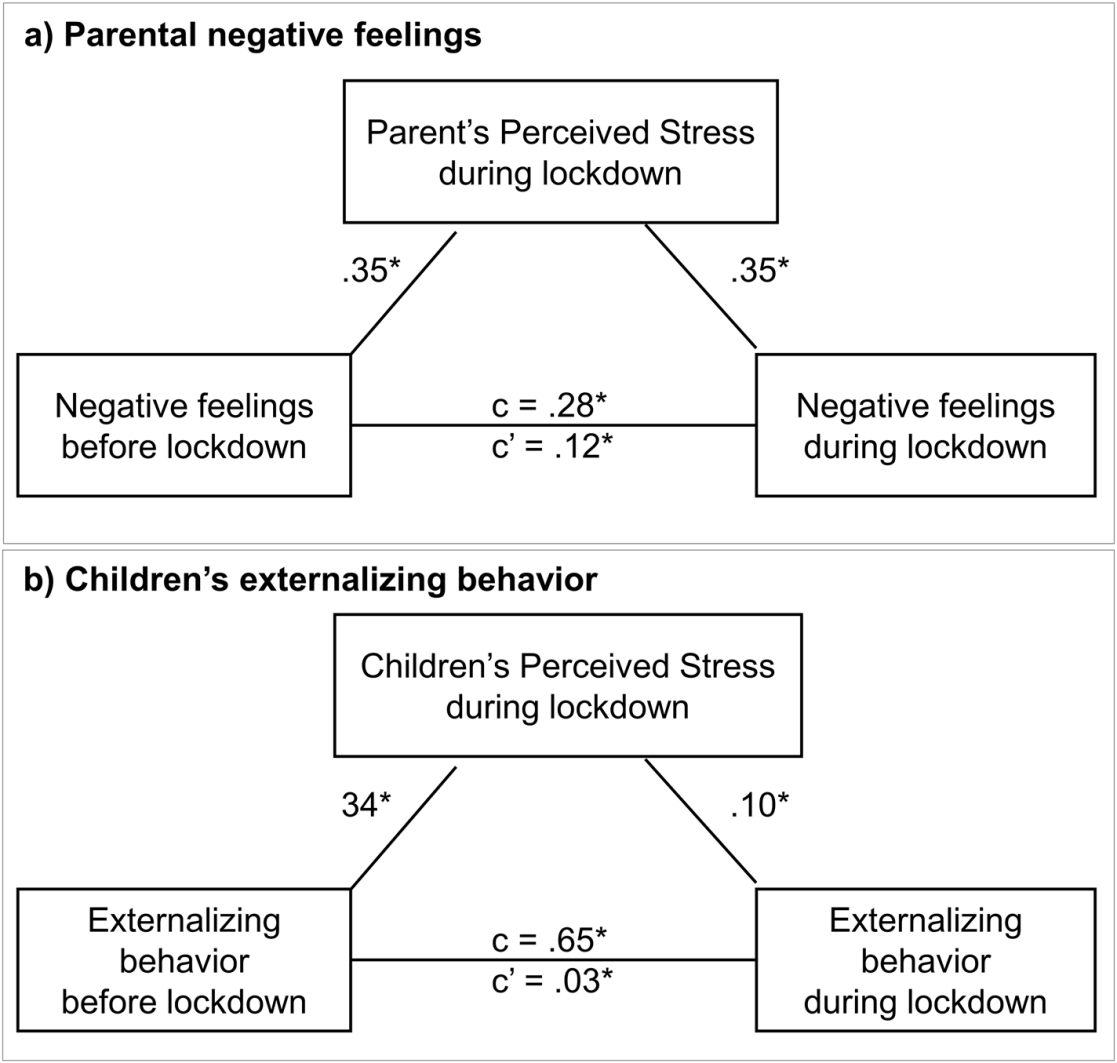
Perceived stress as mediator for longitudinal changes in parental negative feelings (**a**) and externalizing behavior in children (**b**).

The changes in parental negative feelings from before the COVID-19 pandemic lockdown (T4) to during the lockdown (T_COVID_) (path c: *B* = .28, *p* < .001) were mediated by perceived stress during lockdown (path a: *B* = .35, *p* = .003; path b: *B* = .35, *p* < .001; path c’: *B* = .12, 95% confidence interval (CI) .06–.24), see Fig. 4a. Thus, parents with higher levels of negative feelings prior to the lockdown perceived more stress during the lockdown, which resulted in an increase in parental negative feelings during the COVID-19 lockdown.

The changes in externalizing behavior from before the COVID-19 lockdown (T4) to during the lockdown (T_COVID_) were significantly mediated by children’s perceived stress during lockdown (path a: *B* = .34, *p* = .003; path b: *B* = .10, *p* = .037; path c’: *B* = .03, 95% confidence interval (CI) .001 − .08), see Fig. 4b. Thus, children with higher levels of externalizing behavior prior to the lockdown perceived more stress during the lockdown, resulting in an increase in externalizing behavior during the lockdown.

### Factors influencing perceived stress during COVID-19 lockdown

#### Coping strategies

Positive and negative coping strategies were significantly positively associated in both parents (*r* = .22, *p* = .024) and children (*r* = .23, *p* = .006). Parent–child correlations showed a significant association between the child’s and the parent’s negative coping (*r* = .18, *p* = .028), which was not significant for positive coping strategies (*r* = 06, *p* = .503).

For both parents and children, we examined whether positive and negative coping strategies influenced perceived stress, while controlling for confounding variables (age, education level primary parent and number of people in the household during lockdown). We first performed a multiple regression analyses with parental perceived stress as the dependent variable, parental negative and positive coping strategies as independent variables and parental age, parental education level and the number of people in the household during lockdown as confounds. A significant regression was found: *F*(5, 100) = 3.96, *p* = .003, *r*^*2*^ = .17. The individual predictors indicated that parental education (*B* = − .12, *p* = .033); number of people in the household (*B* = .12, *p* = .009); and negative coping strategies (*B* = .24, *p* = .011) were significant predictors of parental perceived stress during lockdown.

Secondly, we performed a multiple regression analyses with children’s perceived stress as the dependent variable, children’s negative and positive coping strategies as independent variables and children’s age, parental education level and the number of people in the household during lockdown as confounds. A significant regression was found (*F*(5, 142) = 11.19, *p* < .001, *r*^2^ = .28. The individual predictors indicated that negative coping strategies (*B* = .57, *p* < .001) were a significant predictor of children’s perceived stress during lockdown.

#### Parental factors influencing children’s stress

Next, we tested whether perceived stress levels of children were influenced by parental factors, i.e., by parental negative feelings and parental overreactivity. As children’s age, parental education level and the number of people in the household during lockdown did not significantly predict children’s stress, we did not include them in the analyses. We first performed a multiple regression analyses with children’s perceived stress as the dependent variable, and parental prior (T4) and current (Tcovid) negative feelings as independent variables. The regression was not significant (*F*(5, 137) = 1.93, *p* = .150), indicating that parental negative feelings did not predict children’s perceived stress during lockdown.

Secondly, we investigated whether prior (T4) and current (Tcovid) parental overreactivity was predictive for children’s perceived stress. A multiple regression analysis was performed with children’s perceived stress as the dependent variable, parental reported prior, parental reported current, and child reported current parental overreactivity as independent variables. The regression was significant: *F*(5, 138) = 6.12, *p* = .001, *r*^2^ = .12. The individual predictors indicated prior parent reported (*B* =  − .21, *p* = .030) and current child reported parental overreactivity (*B* = .22, *p* = .001) were significant predictors of children’s perceived stress during lockdown, whereas current parent reported parental overreactivity was not significant (*B* = .15, *p* = .083).

### Heritability of perceived stress and coping strategies

We took advantage of our unique twin design to explore the genetic and environmental influences on perceived stress and positive and negative coping strategies. Monozygotic twins (n = 41) showed larger within-twin correlations for perceived stress, positive and negative coping than dizygotic twins (n = 26), indicating genetic influences (Table 4). Indeed, behavioral genetic models showed that perceived stress and positive coping strategies were mostly influenced by genetic factors (perceived stress 46%, positive coping 50%, see Table 4). However, negative coping strategies were mostly influenced by shared environmental factors (37%) and showed little heritability (5%). Although these explorative results should be interpreted with caution, as the sample size is rather small for genetic modelling, these results suggest negative coping strategies could be influenced by the shared environment, such as family context, which is in line with the significant association between parents’ and children’s negative coping strategies.

**Table 4:** Within twin associations and estimations of variation in perceived stress, positive coping and negative coping that is explained by heritability (A), shared environment (C) and unique environment (E).

		A	C	E
**Perceived stress**				
r_MZ_	.55*	0.46	0.08	0.45
r_DZ_	.35	[0.00–0.71]	[0.00–0.59]	[0.29–0.70]
**Positive coping**				
r_MZ_	.60*	0.50	0.00	0.50
r_DZ_	− 0.03	[0.24–0.69]	[0.00–0.34]	[0.31–0.76]
**Negative coping**				
r_MZ_	.47*	0.05	0.37	0.58
r_DZ_	.25	[0.00–0.60]	[0.00–0.59]	[0.39–0.81]

N_MZ_ = 41 N_DZ_ = 21 complete twin pairs. Asterisks indicate significant correlations (*p* < .01).

## Discussion

The current COVID-19 pandemic and associated restrictions in terms of social distancing and lockdown can have large impact on families^7^. Specifically, for children in the age range 10 to 13 years who are entering a period of social reorientation, lockdown could have negative consequences^6^. However, the decrease in social and school obligations might be experienced as alleviating, both in children as well as parents^8^. The current study investigated how perceived stress influenced COVID-19 lockdown induced changes in wellbeing of parents and children**.**

### Effects of COVID-19 lockdown on longitudinal trajectories of wellbeing

Overall, we observed relatively low estimates of stress within our sample. Nonetheless, there were significant changes in longitudinal trajectories that can possibly be directly related to the COVID-19 situation. Particularly, we found a strong increase in parental negative feelings (depression, anxiety, hostility and interpersonal sensitivity) during COVID-19 lockdown. This is in line with previous reports that indicated the increased demands on parents, who suddenly have to home school their children, work remotely and might experience excessive worrying about the pandemic situation^11,25^. Despite the increase in parental demand, we also found a slight decrease in parental overreactivity across time. That is, across the whole sample, parents were less overreactive towards their children than two years before. The decrease was also observed before lockdown across a broader time range. Therefore, we cannot conclude whether this decrease is specific for the COVID-19 lockdown situation. Future waves of this ongoing longitudinal study^1^ might provide insights in whether this decrease is pandemic-specific or a more general decrease in parental overreactivity as children grow older and receive more autonomy from their parents^26^.

Longitudinal trajectories of internalizing behavior showed an increase in problem behavior between 2016 (7–9-year-olds) and 2017 (8–10-year-olds), followed by a gradual decrease in problem behavior that seemed decelerated during COVID-19 lockdown. Previous studies also reported an increase in internalizing behavior between age 7 and 10^27^. Notably, we found no significant effects of the COVID-19 lockdown on internalizing behavior in children. That is, we did neither report an increase, nor a decrease, which is in line with previous studies who report stable internalizing behavior across childhood^28,29^. Moreover, there was no strong evidence for changes in externalizing behavior, but there was a marginally significant decrease between 2019 and COVID-19 lockdown. This could indicate that children experience less externalizing behavior, possibly due to less school and sport obligations and more parent–child interaction^8^. However, previous studies have also reported a decrease in externalizing behavior across development^27–30^, which might indicate that the COVID-19 lockdown did not specifically influence externalizing behavior or might have decelerated the developmental decrease. In line with the latter option, a recent paper of Whittle and colleagues reported increased internalizing and externalizing behavior during COVID-19 lockdown in children living in Australia, U.K. and U.S.A^31^.

### Perceived stress as mediator for changes in wellbeing

We aimed to test how perceived stress influenced longitudinal changes in parent and child wellbeing. We specifically focused on the measures that showed COVID-19 specific changes: parental negative feelings and children’s externalizing behavior. We observed that perceived stress was a significant mediator for changes in parental negative feelings. That is, parents who reported higher levels of perceived stress showed stronger increases in anxiety, depression, hostility and interpersonal sensitivity. This in line with a study on COVID-19 lockdown effects in Singapore who also reported a mediating effect of parenting stress^20^. In children we also found a significant mediation effect for perceived stress: children who reported more stress showed a stronger increase in externalizing behavior during the COVID-19 lockdown. These findings again show how prior psychological or behavioral problems might be a risk factor for negative outcomes of the COVID-19 lockdown^10,12,15^.

### Wellbeing and stress during COVID-19 lockdown

Both children and parents indicated that they experienced more free time and more time with the family in the last two weeks, which potentially could serve as a protective factor for stress^8,9,31^. An important and unique aspect of the COVID-19 pandemic is that parents and children are completely reliant on each other, whereas they might typically seek support from their friends or (grand)parents when experiencing stress or negative emotions^32^.

Interestingly and reassuringly, relatively few parents (19%) and children (21%) in our Dutch population sample indicated that stress applied to them in the last two weeks of the COVID-19 lockdown. Our quantitative measure of stress also revealed relatively low estimates of stress in our sample. Similar to what has previously been found in adults^23^, we showed, albeit based on a relatively small sample size for genetic modeling, that perceived stress is heritable, with 46% of the variance in stress being explained by genetic factors. This finding suggests that perceived stress, or resilience towards stress, might be more trait-specific than state-specific^33^. Contrary to what has previously been reported in literature on disasters^13^, perceived stress of children and parents were not significantly correlated, suggesting that increased levels of parental stress are not a risk factor for children’s stress. However, we did find that parental overreactivity was significantly related to children’s perceived stress. These results could indicate that more internally represented stress experiences (perceived stress) of parents do not have a direct effect on children’s stress, whereas external stress-related behaviors such as overreactive parenting increase children’s stress experience. This might provide starting points for parental support programs by showing the importance of dealing with externally represented stress levels during the COVID-19 pandemic.

We hypothesized that positive coping strategies might serve as a protective factor in terms of perceived stress^21,22^. Contrary to our hypothesis, positive coping strategies (i.e., reflection) were not associated to reductions in perceived stress in parents or children. However, in both parents and children, we observed a significant association between negative coping strategies (i.e., rumination) and perceived stress. Twin analyses showed that negative coping strategies were mostly influenced by shared environmental factors (up to 37%). Indeed, parent–child correlations showed a significant association between the parent’s and child’s negative coping. Previous work has also shown the impact of rumination and parental communication during stressful times: difficulty communicating (e.g., not knowing what to say or being too stressed to talk) has been linked to decreased wellbeing in children^34^. A recent study on parent–child communicating during COVID-19 lockdown also showed that parent’s difficulty communicating about COVID-19 with children was associated with increased mental health problems in children^31^, again stressing the adverse effects of rumination and other negative coping strategies.

### Strengths and limitations

The current study made use of an existing longitudinal twin study to study the effects of COVID-19 lockdown on wellbeing of parents and children. Thereby, it moves beyond the mostly theoretical literature on COVID-19 lockdown effects by testing for longitudinal changes due to COVID-19. By combining previously collected data with data collected during the lockdown, we were able to directly test the effects of the lockdown without having to rely on subjective retrospective data.

**Figure 5:**
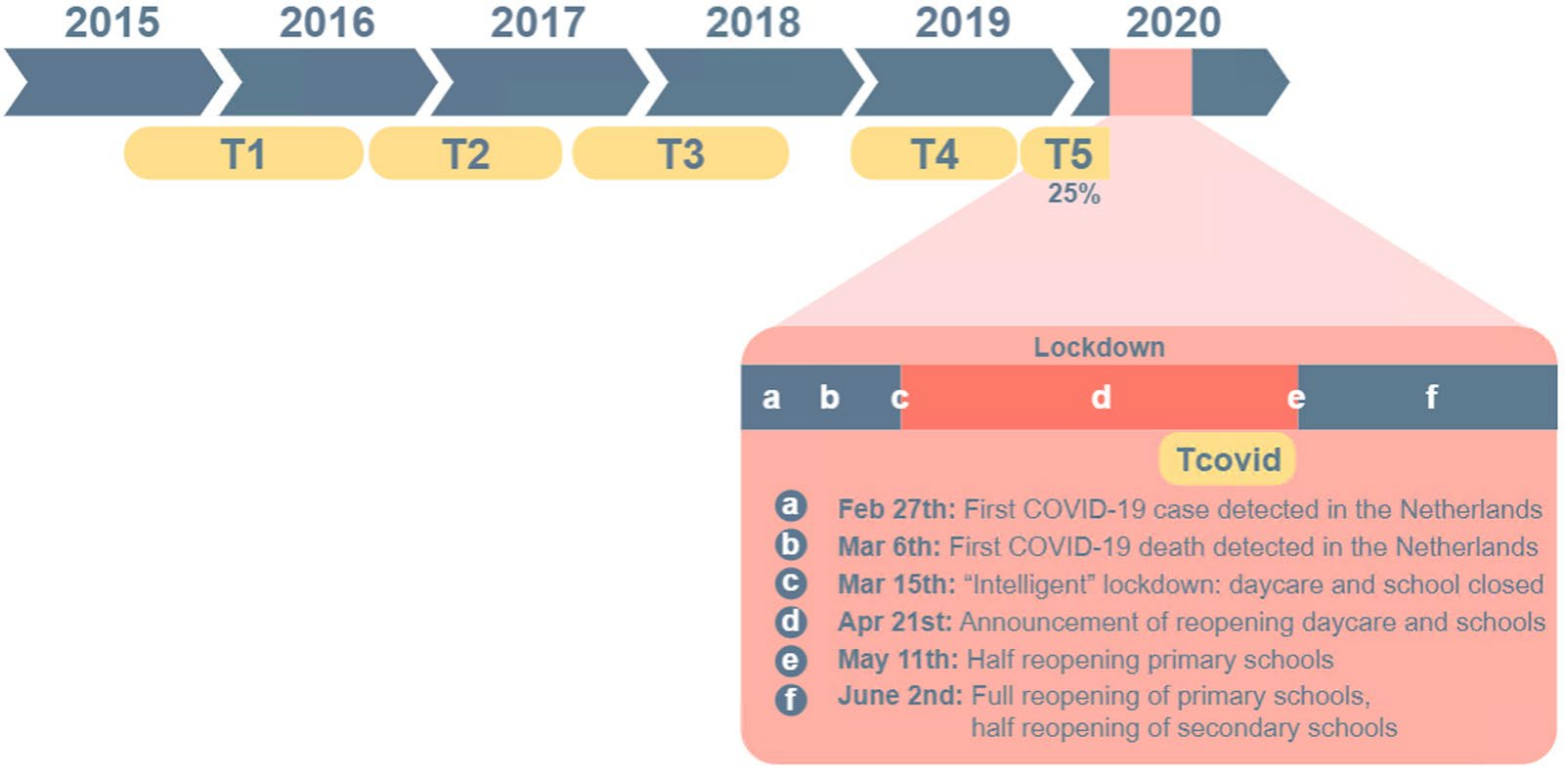
Timeline of data collection and COVID-19 related events in the Netherlands. The data collection of T5 had to be paused due to COVID-19 lockdown. As only 28% of the sample had data on T5 (collected before the COVID-19 lockdown), we did not include these data in the current study. This timeline was created by O.B. using Adobe Illustrator CC 2018, https://www.adobe.com/nl/products/illustrator.html.

Despite these strengths, there are also some important limitations that should be acknowledged. First, the time frame in which parents and children could answer the additional questionnaires during lockdown was rather short (i.e., 2 weeks), as we aimed to finish data collection prior to the reopening of primary schools (see Fig. 5). Despite this, we received a positive response rate of > 50% of the parents that are still included in the L-CID study and of 37% of the children. Nevertheless, due to the stringent time frame, we might have missed out on parents and children who experienced the most stress—as these would not have the time to fill in the questionnaires. However, we found no significant differences in outcome measures (parental negative feelings and overreactivity, and children’s internalizing and externalizing behavior) at T4 between our current sample (responders) and non-responders within the larger L-CID sample (Table 5). Secondly, to limit the work load on our participants in these stressful times we aimed to keep the questionnaires within approximately 30 min response time. Therefore, for some questionnaires we only included a selection of items instead of the complete questionnaire (see Supplements). This can be considered as a limitation as this might affect the validity of the questionnaires used. Last, even though all data was collected prior to reopening the primary schools, parents and children were already aware that the lockdown would be eased. This might have influenced the perceived stress levels of our families such that our data does not display the peak of stress perceived during the lockdown.

**Table 5:** Mean and standard deviations of variables of interest between responders (current sample) and non-responders within the larger L-CID study at time point 4.

Pre-covid (T4)	Responders			Non-responders			Statistics
	N	M	SD	N	M	SD	
Parental negative feelings	101	0.25	0.40	94	0.24	0.41	*t*(193) = 0.13, *p* = .898
Parental overreactivity	98	1.68	0.69	96	1.70	0.67	*t*(197) = 0.05, *p* = .958
Children's externalizing behavior	203	0.42	0.39	250	0.47	0.38	*t*(392) = 1.52, *p* = .129
Children's internalizing behavior	203	0.28	0.35	250	0.27	0.33	*t*(392) = − .29, *p* = .770

## Conclusions

Using the longitudinal twin design of the Leiden Consortium on Individual Development (L-CID^1^) we demonstrated that children in families with (a history of) parental overreactivity might be at risk to experience negative consequences of the COVID-19 lockdown. Moreover, parents and children with relatively lower wellbeing (i.e., more negative feelings in parents and more externalizing behavior in children) prior to the COVID-19 situation experienced more perceived stress during the lockdown, which resulted in even lower wellbeing during lockdown. These results provide important implications for parental support programs, in determining which families might need additional support during the pandemic and thereafter. In a more positive light, we report in general low stress in this Dutch population sample, and group analyses showed stable and even slightly decreasing problem behavior in children. Notably, both parents and children indicated to experience more free time and time with the family. This is in line with recent calls to study potential positive effects of the pandemic^8^ and prior studies on resilience^9^. Future studies should include follow-up measures to truly investigate the long-lasting longitudinal effects of social distancing and lockdown on the development of children and adolescents^6^.

## Methods and materials

### Participants

We invited participants that are enrolled in the ongoing longitudinal twin study of the Leiden Consortium on Individual Development (L-CID^1^) to participate in an additional wave of questionnaire data that specifically focused on the COVID-19 pandemic. The procedures were approved by the Ethical Committee of Psychology (CEP) at the Faculty of Social and Behavioral Sciences at Leiden University and the Dutch Central Committee for Human Research (CCMO). All methods were performed in accordance with the relevant guidelines and regulations of the CEP and CCMO. For the current study we used data of the Middle Childhood Cohort (MCC, see^1^).

**Figure 6:**
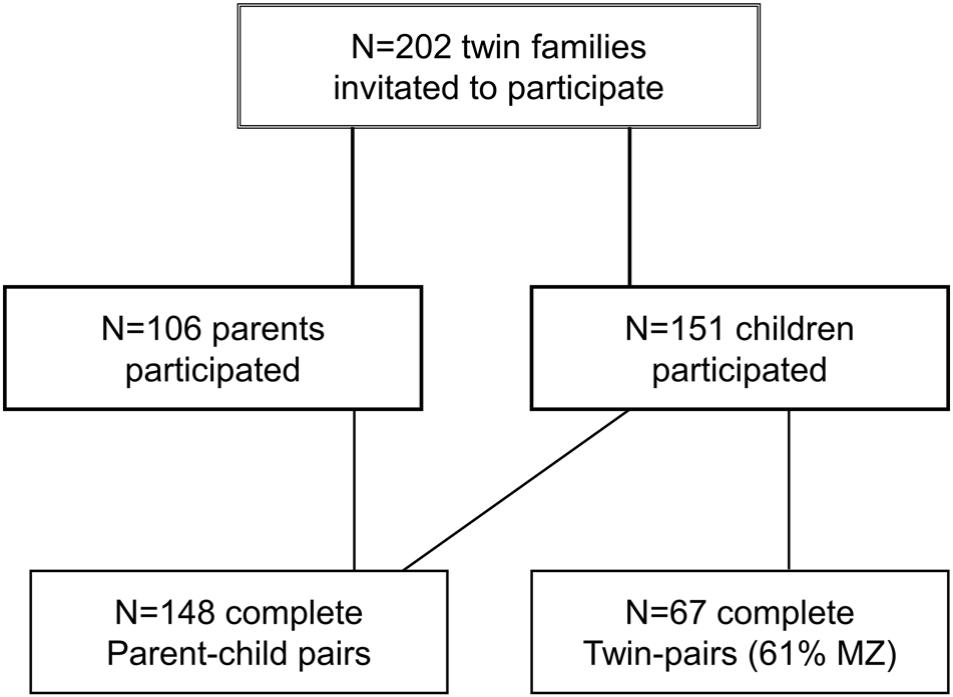
Flowchart of participating parents and children. The contacted families were part of the longitudinal L-CID study of the middle childhood cohort (MCC) and consisted of all families that were enrolled in the L-CID study^1^.

There were 256 families included in the LCID MCC at wave 1 (2015–2016, see^1^ and Fig. 5). In April 2020, 202 families were still enrolled (404 children) and received an invitation. Figure 6 represents a flowchart of the participating primary parents (defined as the parent who spends the most time with the children) and children. A total of 106 primary parents (93% female) agreed to participate by digital informed consent (52% positive response rate). Parents were on average 44.89 years old (SD = 4.97, age range: 33.45–58.80). A total of 151 children (47% girls) agreed to participate (37% positive response rate). As children were less than 18 years old, digital informed consent was obtained from both the child as well as one of their parents. From 148 children (98%) both the child and the primary parent filled in the questionnaires. Children were on average 12.00 years old (SD = 0.81, age range: 10.76–13.65). 68% of the children were enrolled in elementary school and 32% in secondary school. There were 67 twin-pairs where both children completed the questionnaires (61% MZ). The majority of the families were white (89%) and had middle to high socio-economic status (based on parental education: 3% low, 38% middle, 59% high). Parents and children received a digital voucher of €5,- for participating in the study.

There were no significant differences in parental negative feelings, parental overreactivity and children’s internalizing and externalizing behavior at T4 between our current sample (responders) and non-responders within the larger L-CID sample, see Table 5.

### Procedure and timeline

The timeline of data collection and COVID-19 related events are visualized in Fig. 5. A complete overview of the L-CID design and all collected measures is reported in^1^. All questionnaires were digitally administered through Qualtrics. The Dutch “intelligent” lockdown started on March 15th 2020. The Dutch intelligent lockdown contrast with those in most other Western countries as it allowed Dutch citizens more freedom of movement^35^. On April 24th the Dutch prime minister announced reopening of primary schools starting May 11th (see Fig. 5). We sent invitations to participate in the online questionnaire to the primary parents on April 28th 2020. A reminder was sent on May 3rd 2020 and participants could complete the questionnaires until May 10th 2020.

### Longitudinal measures

#### Parental negative feelings

Parents reported on their negative feelings using a shortened version of the Brief Symptom Inventory (BSI^36^) including the subscales depression (6 items), anxiety (6 items), hostility (5 items) and interpersonal sensitivity (4 items). The BSI stated 21 different physical and emotional symptoms (e.g., “*Feelings of worthlessness*”) which were rated on a 5-point scale of distress (0–4), ranging from *‘not-at-all’* to *‘extremely’*. The specific items used are described in the supplements. The BSI has been found to be a valid measure for psychological symptoms both in clinical and non-clinical adult populations^36^. Primary parents completed the questionnaire at T1, T2, T3, T4 and T_COVID_. The Cronbach’s alpha values for the BSI showed good reliability at every time point (α’s > 0.83, see Table 3). Two extreme values were detected at T4, and subsequent analyses were computed with and without these outliers. A mean score was computed based on the 21 items of the BSI. Mean and standard deviations (Table 3) were similar to previously reported non-clinical adult samples^36^ with means ranging between 0.19 and 0.34 on a 0–4 scale. A higher score on the BSI indicates more parental negative feelings.

#### Parental overreactivity

Overreactivity relates to parenting behaviors of irritability, anger, and frustration and is related to harsh or coercive parenting^16^. Overreactive parenting was measured using 10 items of the Parenting Scale**,** on a 7-point Likert scale (PS). The specific items used are described in the supplements. Primary parents completed the questionnaire at T3, T4 and T_COVID_ and filled in the questions for each of the two twin-children separately. The Cronbach’s alpha analyses showed higher reliability if the item about limit setting was removed (see supplements). For the nine-item variant, the PS showed sufficient to good reliability at every time point, for both children (α-range: 0.63–0.80, see Table 3). Pearson’s correlations between parental overreactivity of twin 1 and twin 2 were high (r_T3_ = .81; r_T4_ = .77; r _Tcovid_ = .84, all *p*’s < .001) and therefore we computed a mean score to represent the parent’s overreactivity. At T_COVID_ we also asked the children to report on their parent’s overreactivity, using the same ten items but from the child’s perspective (see the supplements). For the nine-item variant, the child-reported PS showed sufficient reliability (α = 0.70, see Table 3). Parent–child agreement on parental overreactivity during lockdown (T_COVID_) was moderate (*r* = .44, *p* < .001). Data for parental overreactivity showed a normal distribution at every time-point. Mean overreactivity ranged from 1.58 to 1.72 on a 0–6 scale, indicating general low self-reported overreactivity (mean and standard deviations in Table 3).

#### Children’s externalizing and internalizing behavior

Parents reported on the externalizing and internalizing behavior of their children using a shortened version of the Strengths and Difficulties Questionnaire (SDQ^37,38^), including 3 items of each of the five subscales (prosocial; hyperactivity; conduct problems; peer problems; emotional problems). The specific items used are described in the supplements. Primary parents completed the questionnaire at T1, T2, T3, T4 and T_COVID_ and filled in the questions for each of the two twin-children separately. Each item of the SDQ starts with the name of the child, followed by a statement (e.g., *“is restless, overactive and cannot stay still for long”*) which could be rated on a 3-point scale (0–2) ranging from *‘not true’* to *‘certainly true’*. The subscales peer problems and emotional problems were combined to compute a score on internalizing behavior and the subscales hyperactivity and conduct problems were combined to compute a score on externalizing behavior^39^. Table 3 describes the reliability, means and standard deviations for externalizing and internalizing behavior at every time-point. A higher score indicated more internalizing/externalizing problems.

### COVID-19 lockdown measures

#### COVID-19 lockdown related aspects

We provided a list of 20 COVID-19 lockdown related items to examine what parents and children were experiencing during the COVID-19 pandemic lockdown (see Fig. 3). We chose ten positive and ten negative items based on input from the L-CID research team. Parents selected on average 7.75 items (SD = 2.66, range 3–17) and children selected on average 9.52 items (SD = 2.89, range 3–18).

#### Perceived stress

The perceived stress scale (PSS^40,41^) was administered to parents and children as an indication for stress during the COVID-19 lockdown. The PSS is one of the most widely used psychological instruments for measuring the perception of stress^40^. In the current study, we specifically asked about feelings and thoughts during the last two weeks of COVID-19 lockdown. Children and parents completed the 10-item questionnaire using a 5-point scale (0–4) ranging from ‘(almost) never’ to ‘(almost) always’. The Cronbach’s alpha values for the PSS showed sufficient reliability for children (α = 0.68) and parents (α = 0.79). A mean score was computed based on the 10 items. The mean score of PSS was normally distributed in both children and parents. Mean, range and standard deviations of the PSS are described in Table 3. A higher score on the PSS indicated more perceived stress during the lockdown.

#### Positive and negative coping strategies

To obtain an indication of parents’ and children’s coping strategies, we used the short form of the Cognitive Emotion Regulation Questionnaire (CERQ^42^). Participants completed the questionnaire by indicating how often they felt or thought a certain way using a 5-point scale (0–4) ranging from ‘(almost) never’ to ‘(almost) always’. We selected questions from the subscales *Positive reappraisal*, *Positive refocusing* and *Putting into perspective* to compute a score on positive coping strategies (6 items in total). The selected items are described in the supplements. The Cronbach’s alpha values for the positive subscale showed sufficient reliability for children (α = 0.66) and parents (α = 0.73). For negative coping strategies, we selected questions from the subscales *Self-blame*, *Rumination*, and *Catastrophizing* (6 items in total). The selected items are described in the supplements. The Cronbach’s alpha values for the negative subscale showed sufficient reliability for children (α = 0.74) and parents (α = 0.57). Mean, range and standard deviations of positive and negative coping strategies are described in Table 3.

### Statistical analyses

We conducted extensive analyses to shed light on the effects of the COVID-19 lockdown on families by using multiple types of analyses: longitudinal-; moderation and mediation-; multiple regression-; and behavioral genetic analyses. These extensive analyses resulted in multiple comparisons and where applicable we used a Bonferroni correction.

#### Longitudinal analyses

To investigate longitudinal changes in parental negative feelings we used a non-parametric Friedman’s test including data from T1, T2, T3, T4 and T_COVID_. Post-hoc pairwise Wilcoxon tests were used to follow up possible main effects of time. Longitudinal changes in parenting overreactivity were tested using a repeated measures ANOVA including data from T3, T4 and T_COVID_. Post-hoc pairwise comparisons were used to follow up possible main effects of time. To study longitudinal changes in externalizing and internalizing behavior in children we used a non-parametric Friedman’s test including data from T1, T2, T3, T4 and T_COVID_. Post-hoc pairwise Wilcoxon tests were used to follow up possible main effects of time.

#### Moderation and mediation analyses

Moderation and mediation analyses were performed to test whether the longitudinal changes (from T4 to T_COVID_) were influenced by stress during the lockdown. The present study used a bootstrapping approach to moderation and mediation as implemented in the SPSS PROCESS (v3.5) macro of Preacher and Hayes^43^. PROCESS model 1 was selected for moderation analyses (i.e., simple moderation), with prior (T4) parental negative feelings or children’s externalizing behavior as the independent variable, current (Tcovid) parental negative feelings or children’s externalizing behavior as the dependent variable, and the level of perceived stress as the moderator. PROCESS model 4 was selected for mediation analyses (simple mediation), with prior (T4) parental negative feelings or children’s externalizing behavior as the independent variable, current (Tcovid) parental negative feelings or children’s externalizing behavior as the dependent variable, and the level of perceived stress as the mediator. Confidence intervals (95%) were estimated using the bias-corrected bootstrap method (number of resamples = 5000) implemented in the macro.

#### Multiple regression analyses

To investigate which factors influenced perceived stress in parents and children, we performed multiple regression analyses. The assumptions of the multiple regression were met (i.e., normally distributed residuals and independent variables *r* < .80 and variance inflation factor < 10). For both parents and children, we examined whether positive and negative coping strategies (predictors) influenced perceived stress (outcome), while controlling for age, education level of the primary parent and number of people in the household during lockdown (confounds). Next, we investigated which parental factors influenced the level of perceived stress in children. First, we performed a multiple regression analysis where children’s perceived stress (outcome) was predicted by prior (T4) and current (Tcovid) parental negative feelings. We performed a second multiple regression analysis where children’s perceived stress (outcome) was predicted by prior (T4) and current (Tcovid) parental overreactivity.

#### Behavioral genetic analyses

To estimate familial influences on our outcome measures we calculated Pearson within-twin correlations for monozygotic (MZ) and dizygotic (DZ) twin pairs. Zygosity was determined using DNA analyses. DNA was tested with buccal cell samples collected via a mouth swab (Whatman Sterile Omni Swab). Behavioral genetic modeling with the OpenMX package^44^ in R^45^ was used to provide estimates of genetic factors (A), shared environmental factors (C), and unique environmental factors including measurement error (E). The correlation of the shared environment (factor C) was set to 1 for both MZ and DZ twins, while the correlation of the genetic factor (A) was set to 1 for MZ twins and to 0.5 for DZ twins. The last factor, unique environmental influences and measurement error, was freely estimated. We calculated the ACE models for perceived stress and positive and negative coping.

## Supplementary Information

Supplementary Table 1.

Supplementary Table 2.

## Data Availability

Data is publicly available on Dataverse at https://doi.org/10.34894/ZZZEGG.

## Code availability

Code to reproduce all analyses in the manuscript is publicly available on Dataverse at https://doi.org/10.34894/ZZZEGG.
