# Supplementary material for: Perceived stress as mediator for longitudinal effects of the COVID-19 lockdown on wellbeing of parents and children
Source: Sci Rep. 2021 Feb 3;11:2971. doi: 10.1038/s41598-021-81720-8 (PMC7859207; doi:10.1038/s41598-021-81720-8)
Supplement: Supplementary file 1 — Supplementary Table 1. [file 41598_2021_81720_MOESM1_ESM.pdf]

Table S1. Items from the COVID-19 Parent Questionnaire.

**Demographic**		
	1	What is your sex?
	2	Who accompanies the twins during the yearly lab visits?
	3	What are your twins' names?
	4	What is your twins' sex?
	5	What is your highest completed form of education?
	6	Do you experience symptoms of the virus (fever, coughing, sore throat, headache, running nose)?
	7	Do you belong to one of the risk groups to be more prone to being infected with the coronavirus?
	8	Is your profession considered a vital profession?
	9	On average, how many people were living in your house in the past two weeks (including yourself)?
**Perceived Stress Scale**		*The questions in this scale ask you about your feelings and thoughts during the two weeks. For each item, you will be asked to indicate how often you felt or thought a certain way.*
	1	How often have you been upset because of something that happened unexpectedly?
	2	How often have you felt that you were unable to control the important things in your life?
	3	How often have you felt nervous and “stressed”?
	4	How often have you felt confident about your ability to handle your personal problems?
	5	How often have you felt that things were going your way?
	6	How often have you found that you could not cope with all the things that you had to do?
	7	How often have you been able to control irritations in your life?
	8	How often have you felt that you were on top of things?
	9	How often have you been angered because of things that were outside of your control?
	10	How often have you felt difficulties were piling up so high that you could not overcome them?
**Cognitive Emotion Regulation Questionnaire (shortform)**		*Sometimes you experience fun things and sometimes things that are unpleasant When you experience something unpleasant, it can keep your mind occupied for a long time. When you experience something that is unpleasant what are some thoughts you usually have? Read the following sentences and indicate how often you had these thoughts in the past two weeks.*
	1	*I think that I have to accept that this has happened*
negative coping	2	I often think about how I feel about what I have experienced
positive coping	3	I think I can learn something from the situation
negative coping	4	I feel that I am the one who is responsible for what has happened
	5	*I think that I have to accept the situation*
negative coping	6	I am preoccupied with what I think and feel about what I have experienced
positive coping	7	I think of pleasant things that have nothing to do with it
positive coping	8	I think that I can become a stronger person as a result of what has happened
negative coping	9	I continually think how horrible the situation has been
	10	*I feel that others are responsible for what has happened*
positive coping	11	I think of something nice instead of what has happened
	12	*I think about how to change the situation*
positive coping	13	I think that it hasn't been too bad compared to other things
negative coping	14	I think that basically the cause must lie within myself
	15	*I think about a plan of what I can do best*
positive coping	16	I tell myself that there are worse things in life
negative coping	17	I keep thinking about how terrible it is what I have experienced
	18	*I feel that basically the cause lies within others*
**Brief Symptom Inventory**		*The next questions are about your thoughts and feelings in the past two weeks, including today. How often did you experience the following thoughts and feelings?*
anxiety	1	Nervousness or shakiness inside
hostility	2	Feeling easily annoyed or irritated
depression	3	Thoughts of ending your life
anxiety	4	Suddenly scared for no reason
hostility	5	Temper outbursts that you could not control
depression	6	Feeling lonely
depression	7	Feeling blue
depression	8	Feeling no interest in things
anxiety	9	Feeling fearful
interpersonal sensitivity	10	Your feelings being easily hurt
interpersonal sensitivity	11	Feeling that people are unfriendly or dislike you
interpersonal sensitivity	12	Feeling inferior to others
depression	13	Feeling hopeless about the future
anxiety	14	Feeling tense or keyed up
hostility	15	Having urges to beat, injure, or harm someone
hostility	16	Having urges to break or smash things

interpersonal sensitivity	17	Feeling very self-conscious with others
anxiety	18	Spells of terror or panic
hostility	19	Getting into frequent arguments
anxiety	20	Feeling so restless you couldn't sit still
depression	21	Feelings of worthlessness
**Strenght and Difficulties Questionnaire (shortform) ***		*For each item, please mark the box for Not true - Somewhat True- Certainly True. Please give your answers on the basis of how things have been for Child01/Child02 over the last two weeks.*
prosocial	*1*	*Considerate of other people's feelings*
hyperactivity	2	Restless, overactive, cannot stay still for long
conduct problems	3	Often has temper tantrums or hot tempers
peer problems	4	Rather solitary, tends to play alone
conduct problems	5- RE	Generally obedient, usually does what adults request
emotional problems	6	Many worries, often seems worried
prosocial	*7*	*Helpful if someone is hurt, upset or feeling ill*
emotional problems	8	Often unhappy, down-hearted or tearful
hyperactivity	9	Easily distracted, concentration wanders
conduct problems	10	Often lies or cheats
peer problems	11	Picked on or bullied by other children
prosocial	*12*	*Often volunteers to help others (parents, teachers, other children)*
peer problems	13	Gets on better with adults than with other children
emotional problems	14	Many fears, easily scared
hyperactivity	15- RE	Sees tasks through to the end, good attention span
**Parenting Scale ***		*Choose for each statement which button reflects your way of handling situations with Child01/ Child02 the best, for the past two weeks.*
	1	When I'm upset or under stress… I am picky and on my child's back / I am not more picky than usual
	2	*I am the kind of parent that… Sets limits on what my child is allowed to do / Lets my child do whatever he/she wants*
	3- RE	When my child misbehaves… I give my child a long lecture / I keep my talks short and to the point
	4 -RE	When my child misbehaves… I raise my voice or yell / I speak to my child calmly
	5	When I want my child to stop doing something… I firmly tell my child to stop / I coax or beg my child to stop
	6- RE	When there is a problem with my child… Things build up and I do things I don't mean to do / Things don't get out of hand
	7	When my child misbehaves I spank, slap, grab or hit my child… Never or rarely / Most of the time
	8	When my child misbehaves… I handle it without getting upset / I get so frustrated or angry that my child can see I'm upset
	9	When my child misbehaves… I rarely use bad language or curse / I almost always use bad language
	10	When my child does something I don't like, I insult my child, say mean things, or call my child names… Never or rarely / Most of the time
**Wellbeing Semi-Open Questions**		Below some words/terms are displayed that involve the coronacrisis. Select the words/terms that describe your experiences during the past two weeks.
positive	1	Helping others
	2	Fewer obligations
	3	More attention for children
	4	Less schoolwork
	5	Time for (home) hobbies
	6	More time for social media/gaming
	7	Don't get up early
	8	More time with the family
	9	More free time
	10	Uncertainty about the situation
	11	Strict rules for children
	12	Stress
	13	Missing school (activities)
	14	More hassle at home
	15	Strict rules of the government
	16	Can't meet with friends
	17	Less structure
	18	Can't meet grandparents/ family
	19	Missing colleagues
	20	Boredom
