# Supplementary material for: Perceived stress as mediator for longitudinal effects of the COVID-19 lockdown on wellbeing of parents and children
Source: Sci Rep. 2021 Feb 3;11:2971. doi: 10.1038/s41598-021-81720-8 (PMC7859207; doi:10.1038/s41598-021-81720-8)
Supplement: Supplementary file 2 — Supplementary Table 2. [file 41598_2021_81720_MOESM2_ESM.pdf]

Table S2. Items from the COVID-19 Child Questionnaire.

**Demographics**		
	1	What is your sex?
	2	Are you older or younger than your twin?
	3	What grade are you in?
	4	Are you currently experiencing coronavirus symptoms?
	5	Are you currently in home isolation? (this means you don't have any physical contact with the outside world, and that you don't go outside)
	6	On average, how many people were living in your house in the past two weeks (including yourself)?
**Perceived Stress Scale**		*The questions in this scale ask you about your feelings and thoughts during the two weeks. For each item, you will be asked to indicate how often you felt or thought a certain way.*
	1	How often have you been upset because of something that happened unexpectedly?
	2	How often have you felt that you were unable to control the important things in your life?
	3	How often have you felt nervous and "stressed"?
	4	How often have you felt confident about your ability to handle your personal problems?
	5	How often have you felt that things were going your way?
	6	How often have you found that you could not cope with all the things that you had to do?
	7	How often have you been able to control irritations in your life?
	8	How often have you felt that you were on top of things?
	9	How often have you been angered because of things that were outside of your control?
	10	How often have you felt difficulties were piling up so high that you could not overcome them?
**Cognitive Emotion Regulation Questionnaire (shortform)**		*Sometimes you experience fun things and sometimes things that are not so nice. When you experience something not nice, it can keep your mind occupied for a long time. When you experience something that is not nice, what are some thoughts you usually have? Read the following sentences and indicate how often you had these thoughts in the past two weeks.*
	1	*I think that I have to accept that this has happened*
negative coping	2	I often think about how I feel about what I have experienced
positive coping	3	I think I can learn something from the situation
negative coping	4	I feel that I am the one who is responsible for what has happened
	5	*I think that I have to accept the situation*
negative coping	6	I am preoccupied with what I think and feel about what I have experienced
positive coping	7	I think of pleasant things that have nothing to do with it
positive coping	8	I think that I can become a stronger person as a result of what has happened
negative coping	9	I continually think how horrible the situation has been
	10	*I feel that others are responsible for what has happened*
positive coping	11	I think of something nice instead of what has happened
	12	*I think about how to change the situation*
positive coping	13	I think that it hasn't been too bad compared to other things
negative coping	14	I think that basically the cause must lie within myself
	15	*I think about a plan of what I can do best*
positive coping	16	I tell myself that there are worse things in life
negative coping	17	I keep thinking about how terrible it is what I have experienced
	18	*I feel that basically the cause lies within others*
**Parenting scale overreactivity**		*The next questions are about you and your parents.*
primary parent	0	Which one of your parents accompannies you and your twins for the yearly lab visits? [Mother-Father-Stepmother-Stepfather]
	1	When my [] is upset or under stress… He/she is picky and on my back / He/she is not more picky than usual.
	2	*My [] is the kind of parent that… Sets limits on what I am allowed to do / Lets me do whatever I want*
	3- RE	When I misbehave… my [] gives me a long lecture / my [] keeps his/her talks short and to the point
	4- RE	When I misbehave… my [] raised his/her voice or yells / my [] speaks to me calmly
	5	When my [] wants me to stop doing something… He/she firmly tells me to stop / He/she coaxes or begs me to stop
	6- RE	When there is a problem with me… Thing build up and my [] does things he/she doesn't mean to do / Things don't get out of hand
	7	When I misbehave, my [] spanks, slaps, grabs or hits me… Never or rarely / Most of the time
	8	When I misbehave… my [] handles it without getting upset / my [] gets so frustrated or angry that I can see he/she is upset
	9	When I misbehave… my [] rarely uses bad language or curses / my [] almost always uses bad language
	10	When I do something my [] doesn't like, he/she insults me, says mean things or calls me names… never or rarely / most of the time
**Wellbeing Semi-Open Questions**		*Below some words/terms are displayed that involve the coronacrisis. Select the words/terms that describe your experiences during the past two weeks. Select as many/few options as you like.*
positive	1	Helping others
	2	Fewer obligations
	3	More attention from parent(s)
	4	Less schoolwork
	5	Time for (home) hobbies
	6	More time for social media/gaming
	7	Don't get up early
	8	More time with the family
	9	More free time

negative	10	Uncertainty about the situation
	11	Strict rules of parent(s)
	12	Stress
	13	Missing school (activities)
	14	More hassle at home
	15	Strict rules of the government
	16	Can't meet with friends
	17	Less structure
	18	Can't meet grandparents/ family
	19	Missing classmates
	20	Boredom
